# A Successful Heart Transplantation Coupled with Temporary Right Ventricular Assist Device Implantation in a Patient with (ir)Reversible Pulmonary Hypertension

**DOI:** 10.3390/ijerph191912206

**Published:** 2022-09-26

**Authors:** Agnieszka Dyla, Wojciech Mielnicki, Jacek Waszak, Hubert Szurmiak, Krystian Jakimowicz, Roch Pakuła, Michał Oskar Zembala

**Affiliations:** 1 Department of Cardiac Surgery, Cardiac and Lung Transplantation, Mechanical Circulatory Support, Silesian Centre for Heart Diseases, 41-800 Zabrze, Poland; 2 Anaesthesiology and Intensive Care Unit, District Hospital, 55-200 Olawa, Poland; 3 Anaesthesiology and Intensive Care Unit, Specialist Hospital, 38-300 Gorlice, Poland

**Keywords:** heart transplantation, pulmonary hypertension, right ventricle assist device, RVAD

## Abstract

Pulmonary hypertension (PH) constitutes one of the main contraindications to heart transplantation (OHT), and elevated pulmonary vascular resistance (PVR) is associated with high risk of posttransplant right heart failure (RVF). In the present case report, a patient with PH is introduced who qualified for heart lung transplantation (HLT) and underwent successful OHT with temporary right ventricle assist device (tRVAD) due to the lack of a suitable heart-lung donor. Temporary RVAD support coupled with optimal medical management may help reverse pulmonary vascular resistance, which was previously deemed as permanent in patients requiring heart transplantation.

## 1. Introduction

Pulmonary hypertension (PH) constitutes one of the main contraindications to heart transplantation (OHT). Elevated pulmonary vascular resistance (PVR) is associated with a high risk of posttransplant right heart failure (RVF) [1]. Several reports have indicated that in patients with PH-associated with left heart failure, implantation of a left ventricle assist device (LVAD) decreases PVR, facilitating successful bridging to OHT [2,3]. 

## 2. Materials and Methods

Herein, a patient with PH is described who qualified for heart lung transplantation (HLT) and underwent successful OHT with a temporary right ventricle assist device (tRVAD) due to the lack a of suitable heart-lung donor. 

## 3. Case Report

**Figure 1:**
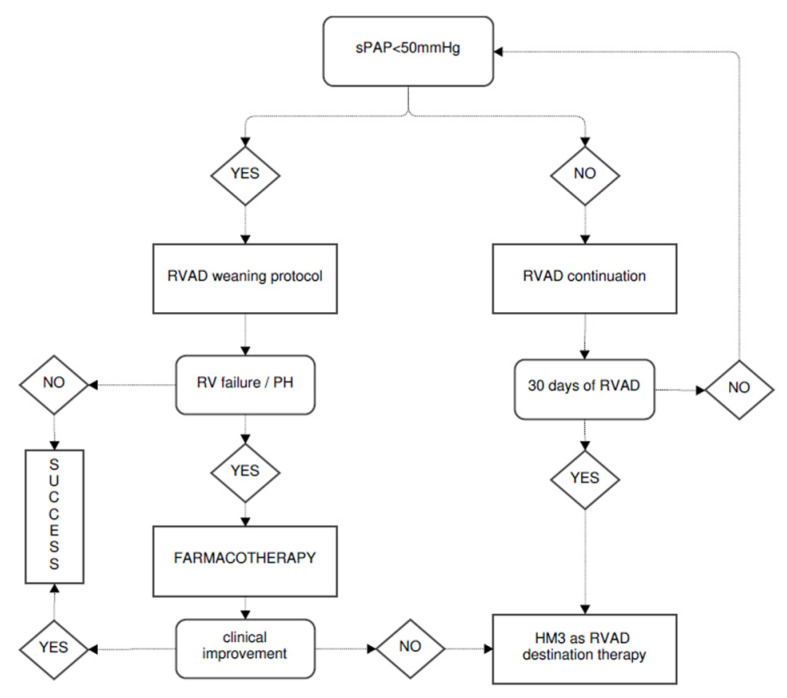
The treatment plan with the tRVAD timeframe.

A 54-year-old woman previously diagnosed with heart failure, possibly due to Lyme disease, was admitted to a hospital with signs of circulatory decompensation. The patient, having already been on optimal medial therapy, was presented to a cardiac transplant team one year ago and did not qualify for OHT because of significant PVR (7.0 WU) with insufficient response to sodium nitroprusside (3.8 WU). Her left ventricular cavity was already severely diminished (34 mm), and its apex was calcified massively, prohibiting LVAD placement. Given the above, the patient was placed on the HLT waiting list and remained hospitalized due to catecholamine dependence. Unfortunately, due to the catecholamine dependence, the patient had to remain hospitalized for the next 6 months. When waiting for the HLT, she experienced intermittent episodes of circulatory deterioration, with the last one occurring in the moment when a matching donor was presented. Unfortunately, the donor’s lungs were not suitable for donation due to infection. After a thoughtful discussion with the patient, the decision was made to proceed with OHT coupled with temporary RVAD support as depicted in Figure 1.

**Figure 2:**
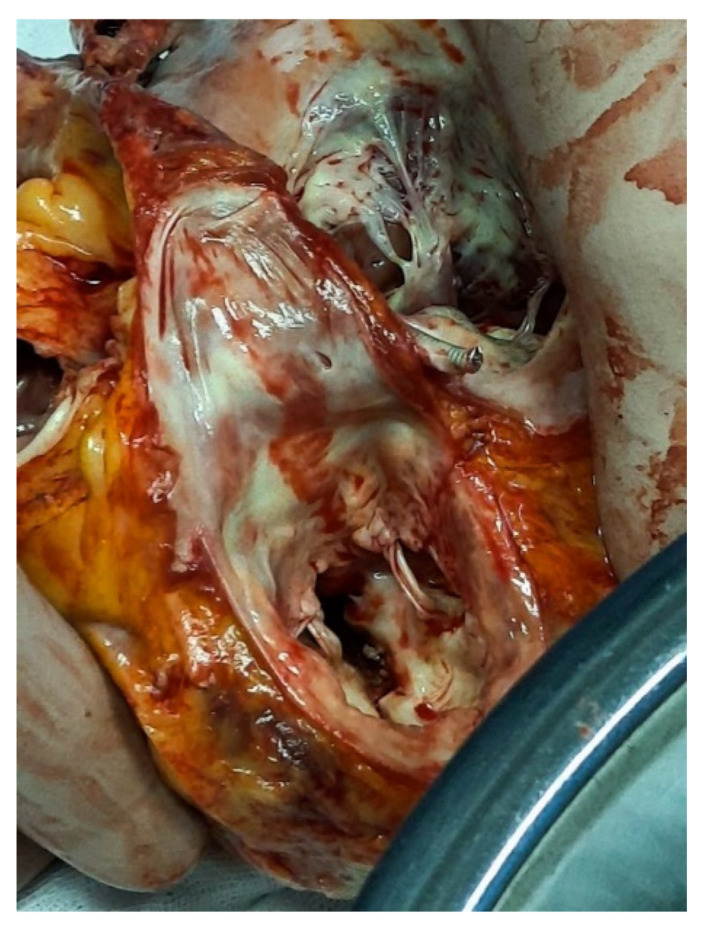
Explanted heart with massive calcification.

OHT was performed using the bicaval technique. A 10mm reinforced graft (Vascutek, Therumo) was sewn proximal to pulmonary trunk’s bifurcation and distal to vascular anastomosis. It was tunneled to an exit point located 3cm below the right costal margin in the mid axillary line, where it was connected to a CentriMag (Abbott Inc., Abbott Park, IL, USA) outflow tubing line. Venous return was facilitated through a multistage femoral cannula (24F) introduced percutaneously at the beginning of the procedure. tRVAD was initiated immediately after extracorporeal circulation ceased and was set to provide blood flow at the rate of 3 liters per minute. Apical calcification proved to be massive, underestimated by imaging tests (Figure 2). 

**Figure 3:**
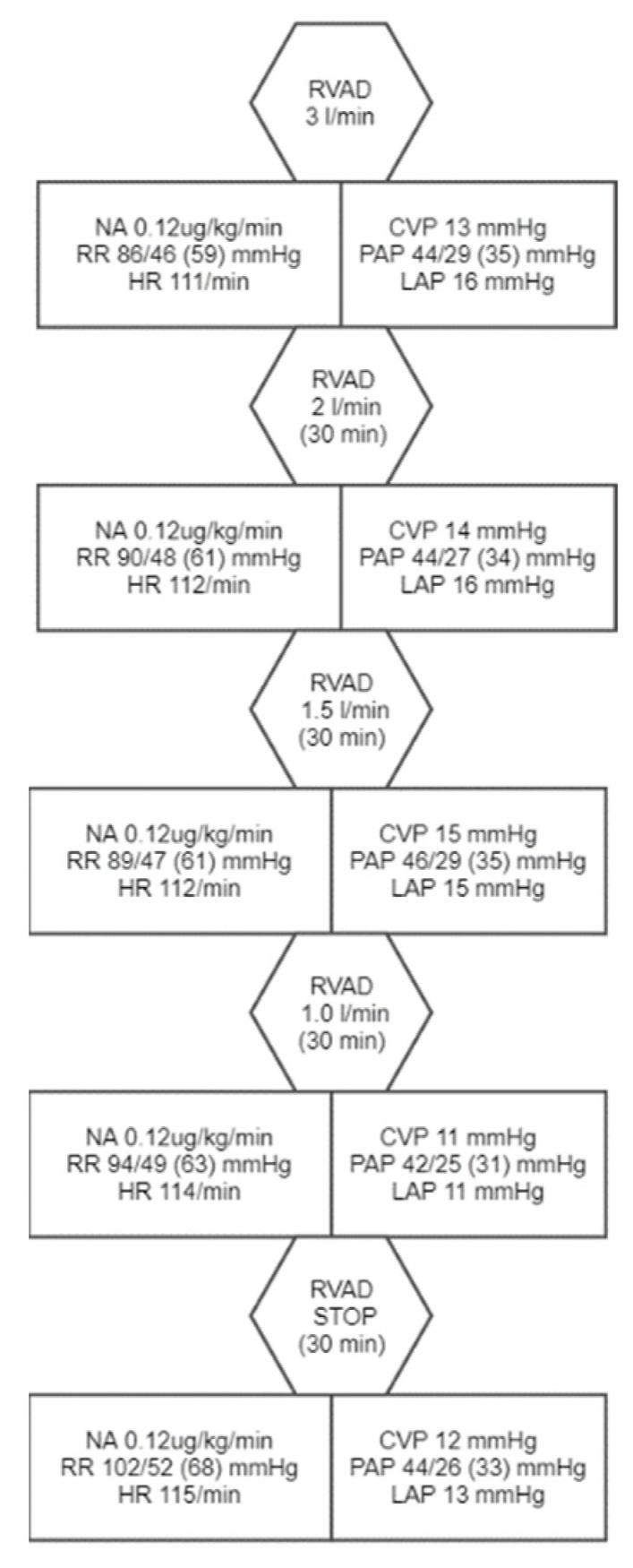
tRVAD weaning protocol with observed vital signs and hemodynamic values. NA—noradrenaline, HR—heart rate, CVP—central venous pressure, PAP—pulmonary arterial pressure, LAP—left atrium pressure.

The patient was quickly extubated and remained hemodynamically stable. Echocardiography presented good LV function, but RV remained overloaded despite adjusted pump flow and sildenafil (3 × 25 mg) treatment. Levosimendan was introduced on the 11th postoperative day (POD). In the first 14 PODs, the patient developed a septic shock due to pneumonia, cardiac tamponade complicated by a cardiac arrest and massive venous congestion in canulated lower extremity. Proper treatment was introduced and allowed for tRVAD removal on the 16th POD, in accordance with the protocol (Figure 3). 

**Figure 4:**
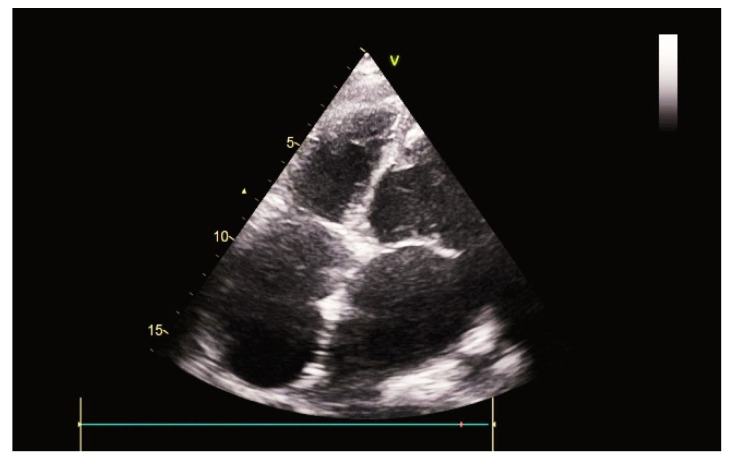
Ultrasound image after tRVAD explantation.

Shortly after RVAD explantation, both RV pressures (92/0–20 mmHg) and PVR (4.5 WU) were elevated and clinically evident, with peripheral oedema and recurring pulmonary infections (Figure 4). 

Renal replacement therapy was used to optimize the circulatory status. Macytentan (1 × 10 mg) and iloprost (6 × 20 mg) were introduced. Meanwhile, severe edema of the lower right extremity evolved into deep venous ulceration and required specialized care. Intensive parenteral nutrition and physiotherapy were carried out continuously.

Three months post OHT, the patient’s condition improved, allowing for a successful discharge on the 123rd POD. Right heart catheterization revealed a complete reversal of PH (RV 72/0–2 mmHg, PVR 1.78 WU), allowing for withdrawal of pharmacotherapy.

## 4. Discussion

Left heart failure is the most frequent cause of PH and an equally important prognostic factor of poor outcome after OHT. The distinction between reversible and irreversible changes in PVR is crucial for OHT candidates [1,3,4,5]. The ISHLT guidelines suggest that sPAP > 60 mmHg, PVR > 5 Wood or TPG > 16–20 mmHg represent a high risk for postoperative RV failure and may result in 30-day mortality [6,7,8,9].

LVAD implantation is a viable option serving as a bridge to OHT, as the pump helps decrease PVR by unloading LV, reducing pulmonary venous pressure, and augmenting cardiac output [2,3]. The most challenging patients are, however, those with biventricular failure or contraindications to LVAD. In such patients, total artificial heart (TAH) or HLT remains the only treatment option [1]. 

The presented case is, to our knowledge, the first published example of this approach and was forced by necessity. Our patient had contraindications to LVAD (massive LV apex calcification), TAH (small chest) and a suitable HLT donor was unavailable despite the nearly 5-month waiting time. During such a long hospitalization, the patient’s condition deteriorated, and she could not be optimized at the time of OHT. We might speculate that performing OHT with tRVAD earlier could have prevented some of the clinical complications (malnutrition, impeded wound healing, infections, muscle thinning). On the other hand, it was notably difficult to come up with a successful solution other than HLT in this particular patient.

The use of tRVAD resembled a technique used in patients in whom LVAD was complicated by acute RV failure [10,11,12]. Lower extremity oedema was clearly associated with peripheral venous cannulation. Although 22F multistage cannula was utilized, it caused unexpectedly significant stasis despite systemic anticoagulation. Central cannulation could be used instead, at a higher risk of bleeding and discomfort, due to it tunneling though the thoracic wall.

The problems with RV in the postoperative period indicated that the process of PH reversal may require a longer duration of tRVAD support, coupled with vasoactive treatment. The simultaneous usage of three different drugs for PH (sildenafil, iloprost, macycentan) could have accelerated the process of PVR normalization. We introduced medical treatment after tRVAD explantation in accordance with the protocol. The question remains, whether simultaneous use of PH treatment and tRVAD, could have prevented RV failure after OHT. Yet, this success led us to believe that PVR irreversibility in severe LVH may not necessarily be irrecusable and could be erroneous. However, further studies are required.

## 5. Conclusions

Temporary RVAD support coupled with optimal medical management may help reverse pulmonary vascular resistance previously deemed as permanent in patients requiring heart transplantation.

## Data Availability

Not applicable.
